# Contraceptive practices and induced abortions status among internal migrant women in Guangzhou, China: a cross-sectional study

**DOI:** 10.1186/s12889-015-1903-2

**Published:** 2015-06-17

**Authors:** Jiazhi Zeng, Guanyang Zou, Xiaoqin Song, Li Ling

**Affiliations:** Faculty of Medical Statistics and Epidemiology, School of Public Health, Sun Yat-sen University, Guangzhou, China; Sun Yat-sen Center for Migrant Health Policy, Sun Yat-sen University, Guangzhou, China; Institute for International Health and Development, Queen Margaret University, Edinburgh, UK

**Keywords:** Contraceptive practices, Induced abortions, Migrant women, Family planning, China

## Abstract

**Background:**

China is facing the unprecedented challenges of internal migration. Migrants tend to have poorer utilization of health and family planning services as compared to the local residents. Migrant women are at greater risk of induced abortions due to their poor contraceptive knowledge and attitude. This study aims to understand the contraceptive practices and history of induced abortions, explore the potential factors influencing induced abortions, and evaluate the utilization of family planning services among migrant women in Guangzhou, China.

**Methods:**

An anonymous, self-administered questionnaire survey was conducted with 1003 migrant women aged 18–49 in Guangzhou, China in 2013. A multi-stage sampling method was employed. Binary logistic regression model was used for analyzing risk factors of induced abortions.

**Results:**

Among the 1003 participants, 810 (80.8 %) reported having sex in the past 6 months, including 715 (88.3 %) married and 95 (11.7 %) unmarried. The most reported contraceptive method was male condom (44.9 %), while 8.1 % never used any contraceptive methods. Only 10.4 % reported having attained free condoms from family planning service stations (FPSSs) and 39.3 % reported having acquired contraceptive knowledge from family planning workers. Of all the participants, 417 (41.6 %) had a history of induced abortion. Of married and unmarried women, 389 (49.1 %) and 28 (14.0 %) had induced abortion respectively. Of these, 152 (36.5 %) had repeated abortions. The most reported reason for having induced abortion was failure of contraception (31.9 %), followed by nonuse of any contraceptives (21.1 %). Migrants who had induced abortion tended to be older, have household registration outside Guangdong province, receive no annual health checkup, have lower education, have urban household registration, have lived longer in Guangzhou and have children (*P* < 0.05).

**Conclusions:**

The prevalence rate of induced abortion, especially repeated abortions among migrant women was high in Guangzhou, China. There is an urgent need to improve the awareness of regular and appropriate use of contraceptives. The utilization of FPSSs among migrant women was reportedly low. Family planning system should be improved to provide better access for migrants and better integrated with the general health services.

**Electronic supplementary material:**

The online version of this article (doi:10.1186/s12889-015-1903-2) contains supplementary material, which is available to authorized users.

## Background

China, the world’s most populous nation, is being challenged by the unprecedented internal migration. In 2013, the number of internal migrants had reached 245 million, more than one-sixth of the total population in China [1]. Since the Reform and Opening up in 1980s, young people have been moving to coastal and urban areas to seek better livelihood [2]. Due to the household registration (hukou) system, migrants are often exploited of many local public services and welfare systems such as medical insurances as resource allocation is mainly based on residents’ local household registration [3, 4].

Sexual and reproductive health problems such as un-planned pregnancies and induced abortions have constituted an important public health problem, especially among female migrants [5]. Migrant women may encounter higher risks of health problems due to the vulnerable socioeconomic status associated with migrants and their inferior identity as female. In China, induced abortion is a legal procedure in the first trimester of pregnancy [6]. China contributed to one fifth of global abortions [7]. Nearly half of the low-income urban women aged 18–49 had experienced induced abortion, while 31 % had experienced repeat abortions [8]. The migrant women, who are often young and middle-aged, are at greater risk of induced abortions due to their poor contraceptive knowledge and attitude [9]. Internationally, induced abortions among immigrant populations were found to be associated with poor socioeconomic status such as poor social networks, poor education and low wages [10, 11]. Evidence is lacking regarding the factors influencing induced abortions among internal migrant women in China. Previous studies have reported the selection and use of contraceptive methods among migrant workers [12]. However, little is known on the factors determining the regular use of contraceptives among migrants.

Family planning system in China relies on a specialized system at the national, provincial, prefectural, county and township levels. Family planning services provide free contraceptives and counseling services for local residents in the family planning service stations (FPSSs). Few initiatives have been conducted which specially focused on rural-to-urban migrants [13]. It was not until in 2010, when the National Population and Family Planning Commission (NPFPC) launched a pilot program to include the migrants into the local family planning services in 49 cities. Guangzhou, where this study was conducted, was one of the pilot cities where free contraceptive tools and counseling services were provided for migrants. So far, it remains unknown to what extent the FPSSs are utilized among migrants. This study aims to understand the contraceptive practices and history of induced abortions, and explore the potential factors influencing induced abortions; and evaluate the utilization of family planning services among migrant women in Guangzhou, China.

## Methods

### Study setting

Guangdong is an economically developed province located in South China. With nearly 30 % of its total population being migrants, this province accommodated the largest number of internal migrants in China [14]. Guangzhou is the capital city of Guangdong province and is one of the largest metropolitans in China. Guangzhou had a population of more than 16 million. More than 50 % of its total population were migrants and among migrants nearly 50 % were female [15]. Baiyun District, where this study was conducted, was one of the ten districts in Guangzhou. This district had the largest migrant population in Guangzhou. Migrants accounted for 50 % of the total population in the district, and more than 25 % of the total migrants in Guangzhou [16].

### Study design

This was a cross-sectional survey conducted in Baiyun District between March and May in 2013. We recruited female migrants1) at childbearing age between 18 and 49; 2) without a local household registration in Guangzhou; 3) having lived in Guangzhou for at least 6 months; 4) providing informed consent. We excluded women with poor literacy, who might have difficulty in understanding and answering the questionnaire even with help of facilitators.

Baiyun District has18 sub-districts; and each district has several community residential committees (CRCs). We recruited the migrants in the CRCs where migrants were supposed to register according to the local policy. Based on the sample size estimation, 79 migrants were needed in each CRC [*n* = *z*_*α*/2_^2^*p*(1 − *p*)/ *δ*^2^ where α was set at 0.05, *z*_*α*/2_ = 1.96, *δ* = 0.10, *p* = 29.0 %, i.e., the induced abortion rate among married women between 20 and 49 years in China [17]]. Considering a loss to follow up rate of 10 %, we aimed to recruit 87 female migrants from each CRC. Due to the constraints of time and funding, 12 CRCs were selected for the study.

A multi-stage sampling method was used. We randomly selected 12 sub-districts from the Baiyun District; from each sub-district one CRC was randomly selected. Each CRC had a registered list of migrants with their names and addresses recorded. We selected the participants in the order they appeared on the list until the sample size was met for each CRC. In total, 1044 eligible migrants were selected from the 12 CRCs.

### Data collection

An anonymous self-administered questionnaire was used and completed by the participants in their residence. The questionnaire was firstly developed based on literature, with consultations sought from experts in health management and reproductive health. A pilot test was conducted with 30 participants in the study district to refine the study variables. The questionnaire was revised accordingly, covering information including: 1) socio-demographic characteristics such as age, marital status, level of education, employment status, and migrant characteristics (e.g., household registration status and years of residence in Guangzhou); 2) contraceptive practices such as contraceptive methods, sources of contraceptives and contraceptive knowledge; 3) history of induced abortions, such as frequencies of pregnancies and induced abortions; 4) general health status, such as annual health checkup and self-rated health status; 5) health insurance status, such as memberships for health insurance and maternity insurance (Additional file 1).

We briefly explained the purpose and content of the study to all the participants and promised to keep their identity and responses confidential before the survey. Small gifts were given to the participants. The postgraduate students from the School of Public Health, Sun Yat-Sen University were trained to conduct the survey. All the completed questionnaires were checked for completeness and consistency.

### Data analysis

Data were double entered into the Epidata 3.1 before being exported to SPSS (USA, 20.0). Descriptive statistics was employed to describe socio-demographic and migrant characteristics, contraceptive practices and induced abortions status of the participants. Means and standard deviations were used for continuous variables, whilst counts and proportions for categorical variables. Univariate analysis such as Chi-square test was used to explore factors influencing frequency of condom use and compare socio-demographic characteristics, migrant characteristics, contraceptive practices and induced abortions status between married and unmarried migrants. The binary logistic regression was employed to explore possible factors associated with induced abortion in the univariate analysis. The dependent variable was induced abortion history (Yes = 1, No = 0). Results were presented as unadjusted odds ratios (ORs) with 95 % confidence intervals (CIs). Then, the binary logistic regression, using step-wise method, was further employed to adjust for potential confounding factors and identify factors that were significantly associated with induced abortion. The independent variables were those with a *p* value < 0.2 in the univariate analysis. Results were presented as adjusted ORs with 95 % CIs. A *p* value <0.05 was considered statistically significant in the analysis.

### Ethics

Written consent was sought from eligible participants. The study was approved by the Ethics Committee of the School of Public health, Sun Yat-sen University, China.

## Results

### Socio-demographic and migrant characteristics

Of the 1044 eligible migrant women, 1020 (97.7 %) consented to participate in the survey. Among them, 1003 (98.3 %) questionnaires were valid. Of the1003 migrant women, most were under 40 (91.0 %), with mean age of 30.7 (±6.2) years. Most were married (80.1 %), educated at or below senior high school (79.4 %), on employment (81.4 %), had household registration outside Guangdong (62.8 %), had rural household registration (71.3 %), had lived in Guangzhou for less than 10 years (70.4 %). As compared to the unmarried group, the married group had higher proportion of migrant women who were above 30 (7.5 % vs.65.6 %, *P* < 0.05), educated at or below senior high school (70.8 % vs.81.5 %, *P* < 0.05), unemployment (1.0 % vs.23.0 %, *P* < 0.05), and resided in Guangzhou for more than 10 years (8.0 % vs.35.0 %, *P* < 0.05) (Table 1).

**Table 1:** Socio-demographic and migrant characteristics among migrant women

Characteristics	Total 1003 (100.0)	Marital status		*χ* ^*2*^	*P*
		Married 803 (80.1)	Unmarried 200 (19.9)		
Age (years)				218.354	<0.001
18~	461 (46.0)	276 (34.4)	185 (92.5)		
30~	452 (45.0)	437 (54.4)	15 (7.5)		
40 ~ 49	90 (9.0)	90 (11.2)	0 (0.0)		
Level of education				44.272	<0.001
Junior high school or less	440 (43.9)	394 (49.0)	46 (23.1)		
Senior high school	356 (35.5)	260 (32.5)	96 (47.7)		
College or more	207 (20.6)	149 (18.5)	58 (29.2)		
Employment status				51.272	<0.001
Employed	816 (81.4)	618 (77.0)	198 (99.0)		
Unemployed	187 (18.6)	185 (23.0)	2 (1.0)		
Household registration place				1.173	0.279
Guangdong province	373 (37.2)	292 (36.4)	81 (40.5)		
Outside Guangdong province	630 (62.8)	511 (63.6)	119 (59.5)		
Household registration type				0.899	0.343
Urban	288 (28.7)	236 (29.4)	52 (26.1)		
Rural	715 (71.3)	567 (70.6)	148 (73.9)		
Years of residence in Guangzhou				122.190	<0.001
≤5	408 (40.7)	259 (32.3)	149 (74.4)		
≤10	298 (29.7)	263 (32.7)	35 (17.6)		
>10	297 (29.6)	281 (35.0)	16 (8.0)		

Note: Married included married, separated and widowed. The number of separated and widowed was 11

### Contraceptive practices

Among the 1003 migrant women, 810 (80.8 %) reported having sexual intercourses in the preceding 6 months, including 715 (88.3 %) married and 95 (11.7 %) unmarried. Of them, 318 (39.3 %) reported having acquired contraceptive knowledge from family planning workers, including 298 (41.7 %) and 20 (21.1 %) of the married and unmarried migrants respectively (*P* < 0.05). The most used two contraceptive methods were reported to be male condom (44.9 %) and intrauterine device (30.9 %), while 8.1 % never used any contraceptive methods. For married migrants, the most used two contraceptive methods were reportedly male condom (41.8 %) and intrauterine device (33.8 %), while 8.1 % never used any contraceptive methods. For unmarried migrant women, the most used contraceptive method was reportedly male condom (68.4 %), while 8.4 % never used any contraceptive methods. Statistical significance was found between married and unmarried women regarding proportion of using male condom, intrauterine device and oral contraceptive (*P* < 0.05). Among the 364 migrants who reported using male condom, 190 (52.2 %) reported using it every time, including 153 (51.2 %) of the married and 37 (56.9 %) of the unmarried; 38 (10.4 %) used it less than half the time, including 31 (10.4 %) of the married and 7 (10.8 %) of the unmarried. Most migrants purchased condoms by themselves and only 38 (10.4 %) attained free condoms from the FPSSs, including 34 (11.4 %) of the married and 4 (6.2 %) of the unmarried (Table 2). The higher education they received, the higher possibility of using condom more than half the time (*P* < 0.05). Migrant women who bought condom themselves tended to use condom more than half the time (*P* < 0.05) (Table 3).

**Table 2:** Contraceptive practices among migrant women who had sex in the past 6 months

Contraceptive practices	Total810 (100.0)	Marital status		*χ* ^*2*^	*P*
		Married715 (88.3)	Unmarried95 (11.7)		
Acquired contraceptive knowledge from family planning workers (*n* = 810)				14.960	<0.001
Yes	318 (39.3)	298 (41.7)	20 (21.1)		
No	492 (60.7)	417 (58.3)	75 (78.9)		
Contraceptive methods (multiple responses allowed) (*n* = 810)					
Intrauterine device (IUD)	250 (30.9)	242 (33.8)	8 (8.4)	25.405	<0.001
Rhythm method/Withdrawal	84 (10.4)	74 (10.3)	10 (10.5)	0.003	1.000
Sterilization	57 (7.0)	49 (6.9)	8 (8.4)	0.315	0.575
Oral contraceptive	26 (3.2)	16 (2.2)	10 (10.5)	18.543	<0.001
Others	8 (1.0)	6 (0.8)	2 (2.1)	1.375	0.241
None	66 (8.1)	58 (8.1)	8 (8.4)	0.011	0.918
Male condom	364 (44.9)	299 (41.8)	65 (68.4)	23.985	<0.001
Frequency of condom use (*n* = 364)				1.425	0.700
Every time	190 (52.2)	153 (51.2)	37 (56.9)		
Most of the time	108 (29.7)	90 (30.1)	18 (27.7)		
Half the time	28 (7.7)	25 (8.4)	3 (4.6)		
Less than half the time	38 (10.4)	31 (10.4)	7 (10.8)		
How to get condom (*n* = 364)				1.540	0.461
From family planning service station	38 (10.4)	34 (11.4)	4 (6.2)		
Bought by self	311 (85.5)	253 (84.6)	58 (89.2)		
Others	15 (4.1)	12 (4.0)	3 (4.6)

**Table 3:** Factors influencing frequency of condom use among migrant women who used male condom

Characteristics	Total364 (100.0)	Frequency of condom use		*χ* ^*2*^	*P*
		More than half the time298 (81.9)	Half or less than half the time66 (18.1)		
Level of education				7.107	0.029
Junior high school or less	127 (34.9)	97 (76.4)	30 (23.6)		
Senior high school	137 (37.6)	111 (81.0)	26 (19.0)		
College or more	100 (27.5)	90 (90.0)	10 (10.0)		
How to get condom				10.478	0.005
From family planning service station	38 (10.4)	25 (65.8)	13 (34.2)		
Bought by self	311 (85.5)	263 (84.6)	48 (15.4)		
Others	15 (4.1)	10 (66.7)	5 (33.3)		
Age (years)				2.085	0.337
18~	189 (51.9)	158 (83.6)	31 (16.4)		
30~	158 (43.4)	128 (81.0)	30 (19.0)		
40 ~ 49	17 (4.7)	12 (70.6)	5 (29.4)		
Marital status				<0.001	0.982
Married	303 (83.2)	248 (81.8)	55 (18.2)		
Unmarried	61 (16.8)	50 (82.0)	11 (18.0)		
Employment status				1.472	0.225
Employed	285 (78.3)	237 (83.2)	48 (16.8)		
Unemployed	79 (21.7)	61 (77.2)	18 (22.8)		
Household registration place				0.218	0.640
Guangdong province	136 (37.4)	113 (83.1)	23 (16.9)		
Outside Guangdong province	228 (62.6)	185 (81.1)	43 (18.9)		
Household registration type				2.350	0.125
Urban	129 (35.4)	111 (86.0)	18 (14.0)		
Rural	235 (64.6)	187 (79.6)	48 (20.4)		
Years of residence in Guangzhou				0.061	0.970
≤5	137 (37.6)	113 (82.5)	24 (17.5)		
≤10	120 (33.0)	98 (81.7)	22 (18.3)		
>10	107 (29.4)	87 (81.3)	20 (18.7)		
Number of children				3.043	0.218
0	90 (24.7)	74 (82.2)	16 (17.8)		
1	207 (56.9)	174 (84.1)	33 (15.9)		
≥2	67 (18.4)	50 (74.6)	17 (25.4)		
Acquired contraceptive knowledge from family planning workers				0.809	0.368
Yes	122 (33.5)	103 (84.4)	19 (15.6)		
No	242 (66.5)	195 (80.6)	47 (19.4)		

### History of induced abortions

Among the 1003 participants, 417 (41.6 %) had a history of induced abortion. Of these, 152 (36.5 %) had repeated abortions, including 110 (26.4 %) had induced abortions twice and 42 (10.1 %) had induced abortions at least three times. For married women, 389 (49.1 %) had induced abortion. Of these, 138 (35.5 %) had repeated abortions. For unmarried women, 28 (14.0 %) had induced abortion. Of these, 14 (50.0 %) had repeated abortions. The most reported reason for induced abortion was failure of contraception (31.9 %), followed by nonuse of contraceptives (21.1 %), risk to maternal/fetal health (17.3 %), disrupt employment (15.6 %) and breaching one-child policy (14.1 %). Of the 417 participants, 119 (30.6 %) married and 14 (50.5 %) unmarried women reported ‘failure of contraception’ as reason for induced abortion, while 79 (20.3 %) married and 9 (31.2 %) unmarried women reported ‘nonuse of contraceptives’. Statistical significance was found between married and unmarried migrants regarding the reason for induced abortion (*P* < 0.05). The most commonly used abortion service venue among the migrants was provincial and municipal hospitals (36.7 %). Only 9.8 % of the migrants used FPSSs, while 6.5 % used private clinics. 60.9 % of the migrants reported having received post abortion care from health facilities (Table 4).

**Table 4:** Induced abortions status among migrant women

Induced abortions status	Total417 (100.0)	Marital status		*χ* ^*2*^	*P*
		Married389 (93.3)	Unmarried28 (6.7)		
Induced abortion times				2.775	0.250
1	265 (63.5)	251 (64.6)	14 (50.0)		
2	110 (26.4)	99 (25.4)	11 (39.3)		
≥3	42 (10.1)	39 (10.0)	3 (10.7)		
Reason for induced abortion				11.752*	0.014
Failure of contraception	133 (31.9)	119 (30.6)	14 (50.0)		
Nonuse of contraceptives	88 (21.1)	79 (20.3)	9 (31.2)		
Risk to maternal/fetal health	72 (17.3)	70 (18.0)	2 (7.1)		
Disrupt employment	65 (15.6)	62 (15.9)	3 (10.7)		
Breaching one-child policy	59 (14.1)	59 (15.2)	0 (0.0)		
Venue for induced abortion				7.481*	0.091
Provincial and municipal hospital	153 (36.7)	142 (36.5)	11 (39.3)		
District hospital	110 (26.4)	100 (25.7)	10 (35.7)		
Township or Sub-district hospital	86 (20.6)	85 (21.9)	1 (3.6)		
Family planning service station	41 (9.8)	38 (9.8)	3 (10.7)		
Private clinic	27 (6.5)	24 (6.2)	3 (10.7)		
Received post abortion care				0.679	0.410
Yes	254 (60.9)	239 (61.4)	15 (53.6)		
No	163 (39.1)	150 (38.6)	13 (46.4)		

Note: Induced abortions status was measured by the last time of induced abortion

*Fisher’s exact test

### Factors influencing induced abortion

Table 5 shows factors influencing induced abortion using binary logistic regression analysis. In the univariate analysis, significant difference was observed between the women who had induced abortion and those who did not have regarding age, household registration place, annual health checkup, level of education, years of residence in Guangzhou, number of children, marital status, employment status, self-rated health status and maternity insurance status (*P* < 0.05). In the multivariate analysis, migrant women aged 30–39 years (OR = 1.654, 95 % CI: 1.158–2.364), from outside Guangdong province (OR = 2.094, 95 % CI: 1.542–2.844) and without annual health checkup (OR = 1.642, 95 % CI: 1.225–2.199) tended to report induced abortion. Migrant women with higher education levels (OR = 0.606, 95 % CI: 0.435–0.843 for senior high school; OR = 0.433, 95 % CI: 0.273–0.687 for college or more), with rural household registration (OR = 0.561, 95 % CI: 0.395–0.795) tended to report fewer induced abortions. Years of residence in Guangzhou (OR = 1.781, 95 % CI: 1.249–2.541 for >5 and ≤10 years; OR = 1.630, 95 % CI: 1.112–2.389 for >10 years) and the number of children (OR = 3.658, 95 % CI: 2.328-5.748 for having one child; OR = 3.697, 95 % CI: 2.184-6.259 for having two or more children) were also significantly associated with induced abortion.

**Table 5:** Binary logistic regression analysis of factors influencing induced abortion among migrant women

Variables	Total1003 (100.0)	Had induced abortion417 (41.6)	Unadjusted *OR* (95 % *CI*)	Adjusted *OR* (95 % *CI*)
Age (years)				
18~	461 (46.0)	120 (26.0)	1.000	1.000
30~	452 (45.0)	243 (53.8)	3.304 (2.501, 4.364)***	1.654 (1.158, 2.364)**
40 ~ 49	90 (9.0)	54 (60.0)	4.262 (2.663, 6.822)***	1.577 (0.914, 2.724)
Household registration place				
Guangdong province	373 (37.2)	104 (27.9)	1.000	1.000
Outside Guangdong province	630 (62.8)	313 (49.7)	2.554 (1.940, 3.362)***	2.094 (1.542, 2.844)***
Annual health checkup				
Yes	606 (60.4)	218 (36.0)	1.000	1.000
No	397 (39.6)	199 (50.1)	1.789 (1.383, 2.314)***	1.642 (1.225, 2.199)**
Level of education				
Junior high school or less	440 (43.9)	235 (53.4)	1.000	1.000
Senior high school	356 (35.5)	127 (35.7)	0.484 (0.363, 0.644)***	0.606 (0.435, 0.843)**
College or more	207 (20.6)	55 (26.6)	0.316 (0.220, 0.453)***	0.433 (0.273, 0.687)***
Household registration type				
Urban	288 (28.7)	132 (45.8)	1.000	1.000
Rural	715 (71.3)	285 (39.9)	0.783 (0.594, 1.032)	0.561 (0.395, 0.795)**
Years of residence in Guangzhou				
≤5	408 (40.7)	111 (27.2)	1.000	1.000
≤10	298 (29.7)	147 (49.3)	2.605 (1.901, 3.569)***	1.781 (1.249, 2.541)**
>10	297 (29.6)	159 (53.5)	3.083 (2.249, 4.226)***	1.630 (1.112, 2.389)**
Number of children				
0	270 (26.9)	34 (12.6)	1.000	1.000
1	472 (47.1)	236 (50.0)	6.883 (4.603, 10.291)***	3.658 (2.328, 5.748)***
≥2	261 (26.0)	147 (56.3)	8.913 (5.769, 13.769)***	3.697 (2.184, 6.259)***
Marital status				
Married	792 (79.0)	389 (49.1)	1.000	
Unmarried	200 (19.9)	28 (14.0)	0.129 (0.081, 0.205)***	-
Employment status				
Employed	816 (81.4)	324 (39.7)	1.000	-
Unemployed	187 (18.6)	93 (49.7)	1.502 (1.092, 2.067)*	-
Self-rated health status				
Excellent/Good	689 (68.7)	258 (37.4)	1.000	-
Fair	297 (29.6)	150 (50.5)	1.705 (1.295, 2.244)***	-
Poor/Very poor	17 (1.7)	9 (52.9)	1.879 (0.716, 4.932)	-
Maternity insurance				
Yes	168 (16.7)	51 (30.4)	1.000	-
No	835 (83.3)	366 (43.8)	1.790 (1.254, 2.556)**	-
Health insurance				
Yes	299 (29.8)	132 (44.1)	1.000	-
No	704 (70.2)	285 (40.5)	0.861 (0.655, 1.131)	-

Note: Variables, except health insurance, had a *P* value <0.2 in the univariate analysis

***P < 0.001, **P < 0.01, *P < 0.05

## Discussion

Our study provides a general picture of contraceptive practices and induced abortions status among migrant women in Guangzhou, China. The prevalence rate of induced abortion was up to 41.6 %, much higher than that of the general population in China (25 %) [18]. The prevalence rate of induced abortion among married migrants and unmarried migrants were 49.1 % and 14.0 % respectively, as compared to 18.0-56.1 % among married non-migrant women [17] and 11.0-55.0 % among unmarried non-migrant women in China [5]. Among migrants who had a history of induced abortion, 36.5 % had repeat abortions. Among unmarried migrants who had induced abortion, 50.0 % had repeat abortions, higher than that of the unmarried non-migrant women in China (33.0 %) [19].

Our study echoes other studies that the predominant reason for seeking abortion among migrant women was contraceptive failure [20]. It indicated that migrant women had taken initiative to avoid unintended pregnancies. However, contraceptive methods might have not been appropriately used, probably due to inadequate counseling and education of contraceptive use provided by family planning services. Another important reason for seeking induced abortion was nonuse of contraceptives. Misperception that using contraceptives could potentially lead to infertility might prevent migrant women from using contraceptives [21, 22]. Our study indicated that premarital sex, which was more likely to be unprotected and resulted in higher risk of unwanted pregnancies and induced abortions, was up to 11.7 % in the past 6 months. Contraceptive use among unmarried migrants remains a matter of great concern. Similar to elsewhere [23], half of the unmarried migrants reported contraceptive failure as the most important reason for the induced abortion. There is an urgent need to improve the use and in particular, proper use of contraceptives among unmarried migrant women.

Our study found that most of the migrants had used general hospitals for induced abortion. Less than 10 % received induced abortion in the FPSSs, suggesting underutilization of the specialized family planning services. About 7 % of the migrants had used the private clinics which were often poorly regulated and unlicensed, and tended to play down the risks and overstate the advantages of abortion [24]. Migrants chose the private clinics probably due to lack of access to the local health insurance and maternal insurance [25], and intention to seek better privacy. In addition, we found about 40 % of the migrants had not received any post abortion care (PAC), which was important to prevent repeated abortions [26, 27]. However, China has not yet fully established formal PAC standards [28]. PAC is urgently needed to be introduced and integrated into hospital and family planning system in China [29].

Our study suggested good awareness of contraception among migrant women. Nearly 92 % of migrants who had sex in the past 6 months used any of the contraceptive methods. Among married and unmarried migrants this was up to 91.9 % and 91.6 %, as compared to 89 % among married non-migrant women and 70 % among unmarried non-migrant women [30]. However, popular use of contraception has not helped to reduce the induced abortion probably due to the high contraceptive failure. As elsewhere [31], condom was the most used method of contraception. However, only half of participants could use it all the time of sexual intercourses. This could be due to poor contraceptive knowledge and attitude among migrant women [9, 32]. Similar to another study [33], we found migrant women with higher level of education were more likely to use condom. We also found that migrant women buying condoms on their own initiative were more likely to have a higher probability of regular condom use than those obtaining free condoms regularly. Further study is needed to understand this pattern, especially on the motivation of condom use.

China has a strong network of family planning services, with major types of contraceptives provided by FPSSs free of charge [24]. Our study suggested the underuse of FPSSs among migrant women, despite migrants in Guangzhou and some other cities have been included into the local family planning services. As elsewhere [32], our study showed that very few (10.4 %) migrant women attained condoms from FPSSs. Less than half migrants, including 41.7 % married migrants and 21.1 % unmarried migrants had acquired contraceptive knowledge from family planning workers. Family planning services specifically targeted unmarried migrants were still absent [34]. Efforts should be made to improve family planning counseling and services, which would increase the effective practice of modern contraceptives [19].

Consistent with findings of another study [35], we found socio-economic and migrant characteristics such as age, education and years of residence in Guangzhou were key determinants of induced abortion among migrant women. Our study also found other determinants such as household registration place, household registration type, annual health checkup and number of children.

Our survey suggested that women with one or more children tended to have induced abortion. Also, migrant women who had an urban household registration were at increased risk of having induced abortion. This may be explained by fear of contravening one-child policy as participants suggested. Under China’s one-child policy, families are allowed to have a second child if the first one is a girl in rural areas, or the couples are only child from their own families. Only very recently has the government loosen the one-child policy, allowing couples to have two children if one spouse is an only child [36]. Future research could explore whether relaxing fertility policy could have impact on reducing abortion among migrants.

A few policy implications can be drawn from this study to improve the family planning of migrant women. Firstly, high prevalence rate of induced abortion, especially repeated abortions among migrant women warrants increased attention from public health authorities to improve effective use of contraceptives and reduce induced abortions. Systematic public education programs should be launched to improve their knowledge and awareness of contraception and abortion. Secondly, the specialized family planning system such as FPSS should improve its coverage for migrants and adopt a comprehensive package of reproductive health services for migrants. These should include enhanced education on reproductive health for young and unmarried migrants, improved supply of contraceptive tools, improved access to free guidance and counseling, and intensified PAC to prevent repeat unplanned pregnancies. Thirdly, it is important to enhance the integration of specialized family planning system and general health services. Chinese family planning system has long been separated from general health services, which turn out to be major venues for induced abortions. The specialized FPSS may take a leading role in providing technical and managerial services, with closer collaboration with general health services. The recent integration of family planning and health management authorities provided great opportunity for close integration at the service level.

Several limitations should be borne in mind. Firstly, report bias may exist in the questionnaire survey. Given the sensitivity of the topic, migrants might feel shy to reveal any private information. However, this problem should be relieved since we used anonymous self-administered questionnaire instead of face-to-face survey. Secondly, a cross-sectional survey did not allow us to explore the causal relationship between variables, though findings from this study could inform future intervention. Thirdly, the generalizability of the findings would be limited as we only conducted the study in one of the districts in Guangzhou. Fourthly, we were not able to identify the empirical relationship between contraceptive practices and induced abortion. We had only surveyed the contraceptive practices among the migrant women who had sex in the recent 6 months, while the induced abortion in their life history. Finally, we did not include non-migrant women for comparison, although literature suggested significant results between these two groups. Further studies which include non-migrant women for comparison will improve the validity of the findings. Despite these limitations, our study provides useful implications for family planning policy development for Guangzhou and other similar cities in China or other emerging countries.

## Conclusions

The prevalence rate of induced abortion, especially repeated abortions among migrant women was high in Guangzhou, China. There is an urgent need to improve the awareness of regular and appropriate use of contraceptives. The utilization of FPSSs among migrant women was reportedly low. Family planning system should be improved to provide better access for migrants and better integrated with the general health services.

## Additional file

Additional file 1:
**Questionnaire for Reproductive Health.** The questionnaire contained three parts: Part 1, Socio-demographic information and health status; Part 2, Questions about contraceptive practices; Part 3, Questions about induced abortions status.
