# Supplementary material for: Contraceptive practices and induced abortions status among internal migrant women in Guangzhou, China: a cross-sectional study
Source: BMC Public Health. 2015 Jun 17;15:552. doi: 10.1186/s12889-015-1903-2 (PMC4469005; doi:10.1186/s12889-015-1903-2)
Supplement: Additional file 1: — Questionnaire for Reproductive Health. The questionnaire contained three parts: Part 1, Socio-demographic information and health status; Part 2, Questions about contraceptive practices; Part 3, Questions about induced abortions status. [file 12889_2015_1903_MOESM1_ESM.docx]

# Additional Files

**Additional file 1 – Questionnaire for Reproductive Health**

**The questionnaire contained three parts: Part 1, Socio-demographic information and health status; Part 2, Questions about contraceptive practices; Part 3, Questions about induced abortions status.**

**Part 1**

1. Age: ____ years old.

2. Education level:

A. Primary school or less B. Junior high school C. Senior high school

D. College or more

3. Employment:

A. Service worker or the sales B. Factory worker C. Self-employed

D. Company employee E. Institution staff F. Housewife G. Unemployed

H. Other, please write in______

4. Marital status:

A. Single B. Married C. Divorced E. Widowed

5. Where is your household registration place?

A. Guangdong province B. Other province, please write in__________

6. What is your household registration type?

A. Rural B. Urban

7. How many years did you live in Guangzhou: ______years

8. Do you have insurance? Where did you buy your insurance?

Health insurance: A. No B. Yes □Guangzhou □Hometown □Other city

Maternity insurance: A. No B. Yes □Guangzhou □Hometown □Other city

9. Do you have health checkup every year?

A. No B. Once C. Twice or more

10. Would you say your health status is?

A. Excellent B. Good C. Fair D. Poor E. Very poor

**Part 2:**

1. Did you have sexual intercourse in the past six months?

A. No (Skip this part) B. Yes

2. How is your living status?

A. Living with husband B. Married but not living with husband

C. Unmarried and living by self D. Cohabitating E. Others

3. Did you acquire any contraceptive knowledge from family planning workers?

A. No B. Yes

4. How did you protect yourself against pregnancy in the past six months? (Multiple choices allowed)

A. Condom (Answer Question 5 and 6) B. Intrauterine device C. Rhythm method

D. Withdrawal E. Sterilization F. Nothing G. Other

5. What was the frequency of condom use?

A. Every time B. Most of the time C. Half of the time D. Less than half the time

6. How did you get condom?

A. From family planning service stations B. Bought by self C. Other

**Part 3**

1. How many children do you have: ____

2. Did you have induced abortion?

A. No (Skip this part) B. Yes. How many times? ____

3. What was the most important reason for your last induced abortion?

A. Failure of contraception B. Nonuse of contraceptives

C. Risk to maternal health D. Risk to fetal health

E. Disrupt employment F. Breaching one-child policy

4. Where did you have the last induced abortion?

A. Provincial and municipal hospital B. District hospital

C. Township hospital D. Sub-district hospital

E. Family planning service station F. Private clinic

5. Did you receive post abortion services after the last induced abortion?

A. No B. Yes
